# The Effects of Traditional Chinese Herbal Dietary Formula on the Ability of Daily Life and Physical Function in Elderly Patients with Mild Cognitive Impairment

**DOI:** 10.3390/brainsci14040333

**Published:** 2024-03-29

**Authors:** Xiaofan Xu, Dan Shi, Yuchen Chen, Luyao Wang, Jiehui Jiang, Shuyun Xiao

**Affiliations:** 1 Department of Brain and Mental Disease, Shanghai Hospital of Traditional Chinese Medicine, Shanghai University of Traditional Chinese Medicine, Shanghai 200444, China; 15055039551@163.com (X.X.); 13042560086@139.com (Y.C.); 2 Yueyang Hospital of Integrated Traditional Chinese Medicine and Western Medicine, Shanghai University of Traditional Chinese Medicine, Shanghai 200444, China; shidan@shutcm.edu.cn; 3 Institute of Biomedical Engineering, Shanghai University, Shanghai 200444, China; wangly1018@shu.edu.cn

**Keywords:** mild cognitive impairment, traditional Chinese herbal dietary formula, daily life ability, physical function

## Abstract

We aimed to examine the association of traditional Chinese herbal dietary formulas with ability of daily life and physical function in elderly patients with mild cognitive impairment. The current study included 60 cases of elderly patients with mild cognitive impairment from Yueyang Hospital of Integrated Traditional Chinese Medicine and Western Medicine, Shanghai University of Traditional Chinese Medicine and Hongkou District, Shanghai. The participants were randomly divided into two groups: group A (herbal dietary formula group, consisting of Alpiniae Oxyphyllae Fructus, Nelumbinis plumula, Chinese Yam, Poria cocos, and Jineijin), 30 cases, and group B (vitamin E), 30 cases, treatment for 3 months. Cognitive function was measured using the Montreal Cognitive Assessment (MOCA) and Mini-Mental State Examination (MMSE); body function was measured using the Chinese Simplified Physical Performance Test (CMPPT), including stand static balance, sitting-up timing, squat timing, and six-meter walk timing. Daily life based on ability was measured by grip strength and the Activity of Daily Living Scale (ADL). The lower the scores of the above items, the poorer the disease degree, except for ADL: the lower the score, the higher the self-care ability. After 3 months of treatment, the two-handed grip strength of both the herbal dietary formula group and vitamin E group increased; the ADL, sitting-up timing, squatting timing, and six-meter walking timing decreased after medication, being statistically significantly different (*p* < 0.05). The two-handed grip strength of group A increased significantly, and the ADL, sitting-up timing, squatting timing, and six-meter walking timing decreased distinctly compared with the vitamin E group. There was a statistically significant difference (*p* < 0.05). The scores of MMSE, MOCA, total CMPPT, and standing static balance of the herbal dietary formula group increased after medication. The difference was statistically significant (*p* < 0.05). The vitamin E group’s MMSE and MOCA scores, CMPPT total scores, and standing resting balance scores did not change significantly after medication (*p* > 0.05). In summary, a traditional Chinese herbal dietary formula can improve body and cognitive function in patients with MCI, and the curative effect is better than that of vitamin E. Traditional Chinese herbal dietary formulas can improve the daily life quality of MCI patients, which has clinical application value.

## 1. Introduction

Epidemiological studies have shown that the prevalence of mild cognitive impairment (MCI) in people over 65 years of age is 10–20% worldwide [1], which will continue to rise with the increase in the proportion of the aged population due to the rapid increase in life expectancy. An important MCI outcome is dementia, and progress to dementia is at a rate of about 10% per year [2]. In 2050, the number of people aged ≥60 years will increase by 1.25 billion, with an estimated 115.4 million persons with dementia [3]. That is why MCI treatment is so important. MCI is a stage that is potentially amenable to interventions that may prevent further decline into dementia. It is estimated that, if the onset of dementia is delayed by 5 years, the overall prevalence of dementia could be reduced by 50%, thereby significantly reducing the burden on care [4].

MCI is a neurodegenerative disease, most of which is a prodromal stage of Alzheimer’s disease (AD). Currently, the clinical pharmacological treatment of MCI mainly refers to AD diagnosis and treatment. Most pharmacological treatment involves cholinesterase inhibitors, represented by donepezil. Other commonly used drugs are ergot alkaloids for protein biosynthesis in the brain and memantine, glutamate receptor antagonists that reduce glutamate concentrations [5]. However, the 2018 American Academy of Neurology (AAN) guidelines pointed out that no high-quality evidence exists to support pharmacologic treatments for MCI [6]. The Chinese guidelines for the diagnosis and treatment of dementia and cognitive impairment [7] also hold that cholinesterase inhibitors that have commonly adverse reactions are ineffective in slowing the progression of dementia in patients with MCI. The guidelines recommend allopathic treatment, which is individualized based on individual differences, including elimination of or reduction in risk factors that promote the development of MCI, and supplementation with nutrients, coinciding with the philosophy of Chinese medicine. Traditional Chinese medicine (TCM), herbs, and derived formulae that promote a healthy state in the body that have been used by Chinese people over a long history to cure diseases may provide broad prospects for drug development for neurodegenerative diseases [8].

MCI belongs to the category of “chi dai” in TCM. TCM holds that “kidney rules wisdom, kidney deficiency means lack of wisdom” [9]; loss of kidney function (shen jing) is accompanied by a lack of nutrients to the brain, which leads to gradual diminishing of the brain and causes dementia [10]. The heart governs the spirit. Heart qi deficiency can lead to forgetfulness and cognitive impairment [11]. The occurrence of MCI is most closely related to heart and kidney deficiency, or because of old age, or due to innate endowment insufficiency resulting in the function of the internal organs being weak. The deficiency of positive qi makes it easy for obscene evils to arise, and the pathological factors are dominated by phlegm dampness. Chengzhi Yang [12] found that the most common symptom in MCI patients is kidney essence deficiency, followed by phlegm turbidity obstruction. Kidney essence deficiency syndrome was significantly correlated with similarity, and phlegm dampness syndrome was correlated with completion of picture and word learning discrimination. Xiangming Deng [13] also confirmed this point in his team’s research regarding the characteristics of TCM constitution for MCI. They found that MCI patients were dominated by the constitution of Yang deficiency, followed by phlegm dampness. Some researchers [14] collected and analyzed clinical research papers on the treatment of MCI with TCM based on data mining software and found that the most common type of MCI is a deficiency in kidney essence with phlegm and blood stasis blocking the collaterals.

We conducted traditional Chinese medicine dialectical analysis of patients with physical examination. Patients with heart and kidney deficiency often have white and dry hair, palpitations, weakness, hearing loss, lumbago and limp legs, difficulty walking, frequent nocturnal urination, dripping urine, sexual function decline, white tongue, weak pulse, and other manifestations. Patients with phlegm turbidity obstruction often have manifestations such as lack of expression, dizziness, fatigue, lethargy, snoring, phlegm, obesity, greasy tongue, and slippery pulse. According to the ideas of “treating the future disease” and “medicine and food are from the same source” in TCM, we put forward the traditional Chinese herbal dietary formula, consisting of Alpiniae Oxyphyllae Fructus, Nelumbinis plumula, Chinese Yam, Poria cocos, and Jineijin. The composition of this dietary formula is based on the doctrine of “returning essence” [15] and the rules of medication in Chinese medicine for the treatment of “chi dai” [16,17], and the selected medicinal and food products are of the same origin. Alpiniae Oxyphyllae Fructus tonifies the kidneys and benefits the essence, Nelumbinis plumula nourishes the heart and calms the mind, Chinese Yam tonifies the lungs, benefits the kidneys, and strengthens the spleen, Poria cocos strengthens the spleen and resolves phlegm, and Jineijin strengthens the spleen, resolves phlegm, and regulates qi. The whole formula takes Alpiniae Oxyphyllae Fructus and Nelumbinis plumula as the principal herbs, Chinese Yam and Poria cocos as the ministers, and Jineijin as the adjuvant to regulate the heart, tonify the kidneys, strengthen the spleen, and resolve phlegm. It facilitates the prevention and treatment of MCI.

On the basis of evaluating the self-care ability and physical function of elderly MCI patients, we conducted a 3-month traditional Chinese medicine diet intervention for elderly MCI patients. If it can be proved that it can maintain or improve the self-care ability and physical function of elderly MCI patients, it can be widely used in clinical practice, which has a very important role in improving the cognitive function of MCI patients, early prevention and intervention regarding MCI and dementia, and improving the quality of life of elderly MCI patients in old age, reducing the burden of caregivers and social function.

## 2. Materials and Methods

### 2.1. Inclusion Criteria and Exclusion Criteria

#### 2.1.1. Inclusion Criteria

(1) Western medical diagnostic criteria for MCI (refer to the 2011 NIA/AA diagnostic criteria for MCI) meeting the following 3 points: (1) cognitive decline: cognitive impairment reported by himself or carer, and evidence of cognitive impairment on objective examination, or cognitive decline confirmed by objective examination. MMSE ≥ 24–27 points, and MoCA < 26 points. (2) Basic daily living skills are normal, with minor impairment of complex instrumental daily living skills. ADL < 26 points. (3) No dementia.
(2) Chinese medicine diagnostic criteria of MCI (refer to Zheng Xiaoyu’s “Guiding Principles for Clinical Research of New Chinese Medicines” from 2002, which refers to dementia in old age with kidney deficiency and medullary decompensation, deficiencies in heart and spleen, and phlegm obstruction, and meets two of the three types of the primary symptoms, and mental degeneration is required. Secondary symptoms with at least two of the three types of secondary symptoms will be included).

#### 2.1.2. Exclusion Criteria

Illiteracy, dementia, cranial CT occupying space or hemorrhage, large infarct foci, impaired consciousness, obvious aphasia, dysarthria, or other serious physical illnesses that could not be cooperated with, combined with liver, kidney, haematopoietic system and endocrine system, and other serious primary diseases and psychiatric patients.

### 2.2. Population under Investigation

The trial was conducted with the written informed consent of all the participants and was approved by Yueyang Hospital of Integrated Traditional Chinese Medicine and Western Medicine hospital institutional ethics committee. Based on the preliminary study, patients had a mean MMSE score of 25.40 with a standard deviation of 2.6. We set the MMSE score of the experimental group to be effective if it was 1.4 higher than that of the control group, and the statistical significance level α = 0.05, β = 0.10, Unilateral test was adopted, and then δ = 1.4, S = 2.6, α = 0.05, β = 0.10; look up the table t_2α_ = 1.645, t_2β_ = 1.282, according to the sample measurement formula *n* = ((t_2α_ + t_2β_)s/δ)^2^, corresponding data *n* = ((1.645 + 1.282)2.6/1.4)^2^ = 29.5 ≈ 30 patients were substituted, and the number of patients in each group was 30. We collected 62 cases of elderly patients with mild cognitive impairment. However, there were 2 patients who did not take medicine regularly, which was recorded as shedding, and the actual number of enrolled cases was 60. The average age of the two groups was 77.6 ± 7.18 for those who used Chinese herbal dietary formula and 76.9 ± 6.73 for those who used vitamin E from Yueyang Hospital of Integrated Traditional Chinese Medicine and Western Medicine, Shanghai University of Traditional Chinese Medicine and Hongkou District, Shanghai. The study patients at the three centers correspond to only one research center, and all included patients were studied by the same group of investigators. The patients’ TCM syndrome type conforms to heart and kidney deficiency with phlegm type, clinical symptoms in accordance with the 2011 NIA/AA diagnostic criteria for MCI [18,19] and the 2002 Guidelines for Clinical Research of New Chinese Medicines for Alzheimer’s Disease [20]: light type of renal deficiency with reduced medulla, light type of deficiency of heart and spleen, light type of phlegm obstruction, and two of the three types of symptoms (light type), and mental degeneration is required; the secondary symptoms have at least two of the three types of secondary symptoms or more at the same time.

### 2.3. Treatment Methods and Procedures

**Figure 1:**
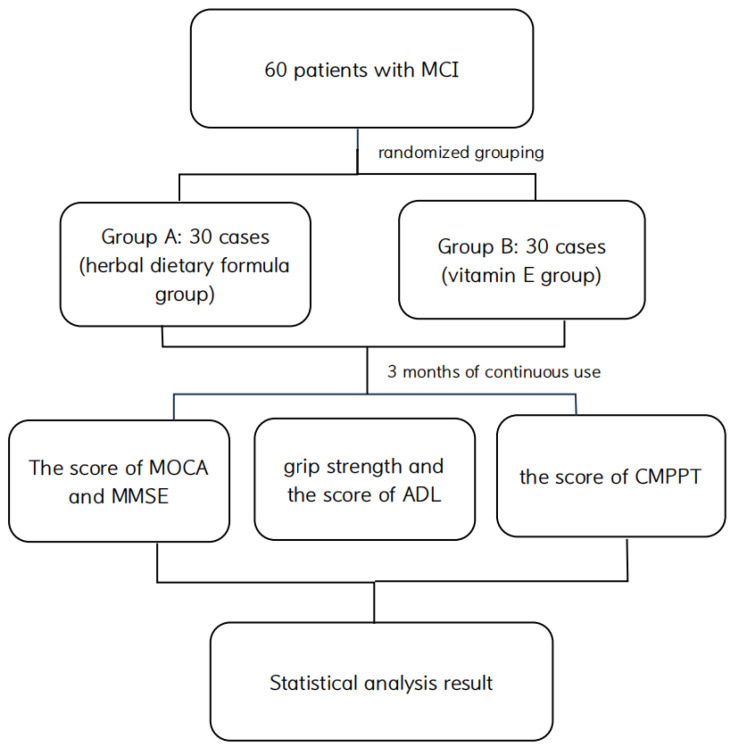
Flow diagram illustrating the study selection process.

Patients were instructed to discontinue medications affecting cognitive function and the central nervous system 2 weeks before and during the trial to exclude interference from other medications before the official start of the clinical trial. Sixty patients with MCI were randomly divided into two groups, A and B. Group A took one packet of Chinese herbal dietary formula (27 g of powder) per day, which consisted of 6 g of Alpiniae Oxyphyllae Fructus, 6 g of Nelumbinis plumula, 5 g of Chinese Yam, 10 g of Poria cocos, and 3 g of Jineijin, while group B took one capsule of vitamin E (100 mg) per day. Both of dietary formula and vitamin E were taken half an hour after lunch and with 150 mL warm water. The effect of the treatment was observed after 3 months of continuous administration. The following is the technical flow chart. Patients were assessed with the Montreal Cognitive Assessment (MOCA) and Mini-Mental State Examination (MMSE), Chinese Simplified Physical Performance Test (CMPPT) [21], including stand static balance, sitting-up timing, squat timing, six-meter walk timing, grip strength, and Activity of Daily Living Scale (ADL) before and after treatment. The study selection process flow diagram as shown Figure 1.

### 2.4. Statistical Analysis

We used SPSS25.0 to enter and analyze the data, using means and standard deviations to indicate measures that conform to normal distribution, and medians (interquartile spacing) to indicate measures that do not conform to normal distribution. Frequencies and percentages were used to indicate count data, and then all data were examined to determine whether they conformed to normality. We used *t*-test to analyze the measurement data to derive the t-value and *p*-value; the count data were analyzed to obtain χ^2^ values and *p*-values using χ^2^ test (all the statistical results obtained were taken as *p*-values less than 0.05 and 0.01 as the indicator of statistical significance).

## 3. Results

### 3.1. Comparison of the Two Groups’ General Information

The general information of the two groups, such as literacy and previous medical history, was tested to be consistent with a normal distribution via normality test, and the χ^2^ test was used. The specific results are displayed in Table 1.

After testing, if the *p*-values of the general information of both groups were greater than 0.05, the difference was not statistically significant and they were comparable.

### 3.2. Balanced Comparison of the Two Groups before Medication

The scores of cognitive function, grip strength, ADL, body function, and TCM symptoms of the two groups were the measurement data, tested by group *t*-test. The specific results are displayed in Table 2.

After testing, if all the *p*-values of both groups were greater than 0.05, the difference was not statistically significant and they were comparable.

### 3.3. Comparison of the Two Groups before and after Medication

The scores of cognitive function, grip strength, ADL, and body function before and after medication of the two groups were used as measurement data, tested by group *t*-test.

After medication, the two-handed grip strength of both groups A and B increased, and the ADL, sitting-up timing, squatting timing, and six-meter walking timing decreased after medication, being statistically significantly different (*p* < 0.05). The two-handed grip strength of group A increased significantly, and the ADL, sitting-up timing, squatting timing, and six-meter walking timing decreased distinctly compared with group B. There was a statistically significant difference (*p* < 0.05). The scores of MMSE, MOCA, total CMPPT, and standing static balance of group A increased after medication. The difference was statistically significant (*p* < 0.05). Group B’s MMSE and MOCA scores, CMPPT total scores, and standing resting balance scores did not change significantly after medication (*p* > 0.05). The specific results are displayed in Table 3.

Our findings indicate that the herbal dietary formula can improve the grip strength, cognitive function, physical function, and ability of daily living in patients with MCI, and the efficacy is better than vitamin E.

## 4. Discussion

Research shows that both TCM compounds and extracts can act on multiple targets and pathways involved in the complex pathogenesis of cognitive dysfunction [22]. The traditional Chinese herbal dietary formula consists of Alpiniae Oxyphyllae Fructus, Nelumbinis plumula, Chinese Yam, Poria cocos, and Jineijin. Alpiniae Oxyphyllae Fructus has the effect of strengthening the kidneys and warming the spleen. Modern pharmacological studies have shown that many active components of Alpiniae Oxyphyllae Fructus have definite neuroprotective effects. It demonstrates anti-inflammatory, anti-oxidative stress damage, inhibition of Aβ protein deposition, inhibition of acetylcholinesterase and Tau phosphorylation, improvement of mitochondrial dysfunction, and promotion of cell migration, regeneration, and proliferation effects [23]. Nelumbinis plumula has the effect of tonifying the spleen, benefiting the kidneys, and nourishing the heart. Modern pharmacological studies have shown that Nelumbinis plumula can influence antioxidants, free radical scavenging, inspire cholinergic synapses, and calm the nerves [24]. Chinese Yam can tonify the spleen and stomach and nourish kidney and lung function. Modern pharmacological studies have found that it can have anti-inflammatory, antioxidant, anti-tumor, and immune regulation effects and promote kidney regeneration and repair [25]. Poria cocos has the effect of inducing diuresis and seepage of dampness, strengthening the spleen, and tranquilizing the heart. Modern pharmacological studies have suggested that it demonstrates anti-inflammatory, antioxidant, anti-tumor, and immune regulation effects and serves as a liver protection diuretic [26]. Jineijin has the effect of stomach digestion, and astringent essence stops negative effects: modern pharmacological studies show that it has the functions of regulating the gastrointestinal tract, reducing oxidative stress, anti-calculus, anticoagulation, and improving hemorheology [27]. The above five herbs, when used together, can regulate the heart and tonify the kidneys, strengthen the spleen, and expel phlegm, which can effectively improve clinical symptoms in patients with MCI.

As MCI is a complicated and multifactorial disease, Chinese medicine offers the advantage of modulating the systemic organs via synergetic effects of prescription compatibility and different compositions within a single herb [10]. Recent research [28] found that autophagy is the main process by which misfolded abnormal aggregation of diverse proteins, which is one of the common features of neurodegenerative diseases, and damaged organelles are removed from cells, and dysregulation of autophagy leads to the formation of amyloid β peptide (Aβ), the hallmark of AD. The pharmacological activation of autophagy may be beneficial to regulate neurodegenerative diseases. Studies have reported the identification of novel autophagic enhancers from TCMs as potential neuroprotective agents [10], such as Nelumbinis plumula, the monarch drug in the traditional Chinese herbal dietary formula. Research has also found that factors that seem to have no direct relationship to the brain, such as hyperhomocysteinemia, midlife hypertension, etc., can certainly affect disease progression, which may explain why herbs’ general health promotion effects can eventually benefit the aging brain [29].

The following problems exist in this study: (1) sample size: due to time constraints, the sample size of this study is on the small side. It is necessary to expand the sample size to continue this research in the future. (2) Experimental time: the research time is relatively short. It will be continued in the future. (3) Adherence problems: most of the subjects came from outpatient clinics and the community. Adherence needs to be further supervised. (4) Action mechanism: the modern pharmacologic mechanism of action of the dietary formula for MCI has not been further investigated. (5) Taste and adverse reactions: due to the bitter taste of this recipe, some patients suffering from stomach disorders may experience nausea and vomiting.

## 5. Conclusions

In conclusion, we found that cognitive function is related to daily living ability and physical function, and grip strength is related to daily living ability. Good grip strength and somatic function can improve the daily living ability of elderly patients with MCI, which in turn improves the cognitive function of elderly patients with MCI. The traditional Chinese herbal dietary formula can improve the grip strength and somatic function of MCI patients in order to slow down the deterioration of patients’ daily life ability and improve their self-care ability. Our study emphasized that traditional Chinese herbal dietary formula use is an effective method to improve cognitive function and physical function in elderly patients with mild cognitive impairment.

## Figures and Tables

**Table 1:** Comparison of remaining general information between two groups.

Item	Category	Group A		Group B		χ^2^	*p*
		*n* = 30	%	*n* = 30	%		
Gender	Male	15	50.0	13	43.3	0.268	0.605
	Female	15	50.0	17	56.7		
Literacy level	Illiterate	5	16.7	4	13.3	0.005	0.946
	Primary School	5	16.7	5	16.7		
	Junior High	5	16.7	6	20.0		
	High school	7	23.3	8	26.7		
	Technical secondary school	1	3.3	1	3.3		
	Junior College	4	13.3	2	6.7		
	University	3	10.0	4	13.3		
Diabetes	Yes	6	20.0	4	13.3	0.480	0.488
	No	24	80.0	26	86.7		
Coronary heart disease	Yes	7	23.3	4	13.3	1.002	0.317
	No	23	76.7	26	86.7		
Arrhythmia	Yes	11	36.7	13	43.3	0.278	0.598
	No	19	63.3	23	56.7		
Hypertension	Yes	12	40.0	10	33.3	0.287	0.592
	No	18	60.0	20	67.7		
Hyperlipidemia	Yes	4	13.3	2	6.7	0.185	0.667
	No	26	86.7	28	93.3		
Cerebral infarction	Yes	6	20.0	3	10.0	0.523	0.470
	No	24	80.0	27	90.0		
Smoking history	Yes	6	20.0	8	26.7	0.373	0.542
	No	24	80.0	22	73.3		

**Table 2:** Balanced comparison between two groups before medication.

Item	Group A (M ± SD)	Group B (M ± SD)	t	*p*
MMSE	25.40 ± 2.55	25.57 ± 2.25	−0.268	0.789
MOCA	17.42 ± 2.29	17.77 ± 2.83	−0.526	0.601
ADL	18.90 ± 2.63	19.20 ± 2.79	−0.428	0.670
Left grip strength	22.93 ± 3.48	22.53 ± 3.01	0.476	0.636
Right grip strength	23.03 ± 3.26	23.40 ± 3.68	−0.408	0.685
Total CMPPT scores	10.00 ± 1.02	9.83 ± 0.82	0.559	0.578
Stand static balance scores	2.83 ± 0.83	2.87 ± 0.82	−0.156	0.876
Sitting-up timing	15.20 ± 2.91	15.33 ± 2.26	−0.198	0.844
Squat timing	3.08 ± 0.64	2.93 ± 0.49	1.023	0.311
6 m walk timing	10.37 ± 1.42	10.93 ± 1.25	−1.632	0.108

**Table 3:** Comparison between group A and group B before and after medication.

Item	Pre-Treatment (M ± SD)		Post-Treatment (M ± SD)		t	*p*
	Group A	Group B	Group A	Group B		
MMSE	25.40 ± 2.55	25.57 ± 2.25	27.10 ± 1.72 *	25.83 ± 2.20	2.448	0.017
MOCA	17.42 ± 2.29	17.77 ± 2.83	20.28 ± 2.10 *	18.88 ± 2.62	2.292	0.026
ADL	18.90 ± 2.63	19.20 ± 2.79	16.53 ± 2.22 *	18.13 ± 2.57 *	2.579	0.012
Left grip strength	22.93 ± 3.48	22.53 ± 3.01	25.61 ± 2.72 *	24.10 ± 2.97 *	2.037	0.046
Right grip strength	23.03 ± 3.26	23.40 ± 3.68	26.27 ± 3.35 *	24.71 ± 2.42 *	2.070	0.043
Total CMPPT scores	10.00 ± 1.02	9.83 ± 0.82	11.10 ± 0.88 *	10.13 ± 0.78	4.499	<0.001
Stand static balance scores	2.83 ± 0.83	2.87 ± 0.82	3.46 ± 0.63 *	3.13 ± 0.73	1.894	0.063
sitting-up timing	15.20 ± 2.91	15.33 ± 2.26	10.63 ± 2.23 *	14.06 ± 2.99 *	5.033	<0.001
Squat timing	3.08 ± 0.64	2.93 ± 0.49	2.06 ± 0.77 *	2.81 ± 0.65 *	4.044	<0.001
6 m walk timing	10.37 ± 1.42	10.93 ± 1.25	8.27 ± 1.20 *	10.33 ± 0.92 *	7.741	<0.001

Note: t value is the comparative statistical value of AB group after treatment. * indicates that, compared with pre-treatment, *p* < 0.05, the difference was statistically significant.

## Data Availability

The data presented in this study are available on request from the corresponding author. The data are not publicly available due to privacy restrictions.

## References

[1] Xue J., Li J., Liang J., Chen S. (2018). The Prevalence of Mild Cognitive Impairment in China: A Systematic Review. Aging Dis..

[2] Bajwa R.K., Goldberg S.E., Van der Wardt V., Burgon C., Di Lorito C., Godfrey M., Dunlop M., Logan P., Masud T., Gladman J. (2019). A randomised controlled trial of an exercise intervention promoting activity, independence and stability in older adults with mild cognitive impairment and early dementia (PrAISED)—A Protocol. Trials.

[3] Fonte C., Smania N., Pedrinolla A., Munari D., Gandolfi M., Picelli A., Varalta V., Benetti M.V., Brugnera A., Federico A. (2019). Comparison between physical and cognitive treatment in patients with MCI and Alzheimer’s disease. Aging.

[4] Xu Y., Zhu J., Liu H., Qiu Z., Wu M., Liu J., Wu J., Huang J., Liu Z., Liu W. (2023). Effects of Tai Chi combined with tDCS on cognitive function in patients with MCI: A randomized controlled trial. Front. Public Health.

[5] Yang Z.J. (2020). Progress of research on combined Chinese and Western medicine treatment of mild cognitive impairment. J. Pract. Tradit. Chin. Intern. Med..

[6] Petersen R.C., Lopez O., Armstrong M.J., Getchius T.S.D., Ganguli M., Gloss D., Gronseth G.S., Marson D., Pringsheim T., Day G.S. (2018). Practice guideline update summary: Mild cognitive impairment: Report of the Guideline Development, Dissemination, and Implementation Subcommittee of the American Academy of Neurology. Neurology.

[7] Jia J.P. (2016). Chinese Guidelines for the Diagnosis and Treatment of Dementia and Cognitive Impairment.

[8] Zhang J., Yang C., Wei D., Li H., Leung E.L., Deng Q., Liu Z., Fan X.X., Zhang Z. (2019). Long-term efficacy of Chinese medicine Bushen Capsule on cognition and brain activity in patients with amnestic mild cognitive impairment. Pharmacol. Res..

[9] Yao W., Zhao Y., Han X. (2019). Treating Mild Cognitive Impairment from Five Zang Organ. Henan Tradit. Chin. Med..

[10] Law B.Y.K., Wu A.G., Wang M.J., Zhu Y.Z. (2017). Chinese Medicine: A Hope for Neurodegenerative Diseases?. J. Alzheimers Dis..

[11] Wang J., Lin S.M. (2013). Thoughts on the treatment of mild cognitive impairment by regulating heart. Henan Tradit. Chin. Med..

[12] Yang C.Z., Zhong J., Zhu A.H., Tian J.Z. (2003). Study on the Syndrome of Senile Minor Cognitive Injury. J. Beijing Univ. Tradit. Chin. Med..

[13] Deng X., Teng J., Nong X., Yu B., Tang L., Liang J., Zou Z., Liu Q., Zhou L., Li Q. (2021). Characteristics of TCM Constitution and Related Biomarkers for Mild Cognitive Impairment. Neuropsychiatr. Dis. Treat..

[14] Zang M.X., Ding M.R., Zhang T., Qu Y.J., An H.M. (2022). Medication regularity of traditional Chinese medicine for mild cognitive impairment: Based on data mining. Pharmacol. Clin. Chin. Mater. Medica.

[15] Chen C. (2012). Lin Shuimiao’s Academic Thought of “Returning Essence”: A Preliminary Study. J. Tradit. Chin. Med. Lit..

[16] Huang X.F., Zhang T., Zhou Y. (2019). Analysis of medication patterns in the treatment of dementia based on the Chinese medicine inheritance support system. Med. Diet Health.

[17] Zhou J.J., Tan Z.H. (2021). Using data mining to explore the pattern of Chinese medicine use in Alzheimer’s disease. World Chin. Med..

[18] Winblad B., Palmer K., Kivipelto M., Jelic V., Fratiglioni L., Wahlund L.O., Nordberg A., Bäckman L., Albert M., Almkvist O. (2004). Mild cognitive impairment beyond controversies towards a consensus: Report of the International Working Group on Mild Cognitive Impairment. J. Intern. Med..

[19] Portet F., Ousset P.J., Visser P.J., Frisoni G.B., Nobili F., Scheltens P., Vellas P., Touchon J. (2006). Mild cognitive impairment(MCI)in medical practice: A critical review of the concept and new diagnostic procedure. Report of the MCI Working Group of the European Consortium on Alzheimer’s Disease. J. Neurol. Neurosurg. Psychiatry.

[20] Zheng X.Y. (2002). Guiding Principles for Clinical Research of New Chinese Medicines.

[21] Chen D.W., Chen J.W., Du W.J. (2011). Development of the Simple Somatic Performance Test and its reliability. Chin. J. Gerontol..

[22] Pei H., Ma L., Cao Y., Wang F., Li Z., Liu N., Liu M., Wei Y., Li H. (2020). Traditional Chinese Medicine for Alzheimer’s Disease and Other Cognitive Impairment: A Review. Am. J. Chin. Med..

[23] Zhao C.B., Wang J., Qi C.Y., He S.R., Wu X., Wu J.H., Wang C.L. (2021). Research progress on processing of Arisaematis Rhizoma Preparatum. Pharmacol. Clin. Chin. Mater. Medica.

[24] Jiang Y.P., Chen Z.Y., Mo F., Liu M., Huang Q., Liu S. (2019). Pharmacological mechanism of traditional sedative effect of alkaloids in Nelumbinis Plumula based on network pharmacology. China J. Chin. Mater. Medica.

[25] Yang Y.Y., Sun Y.L., Sun J.M., Tang Z.X. (2022). Progress on pharmacological effects of active ingredients in Chinese. Chin. Wild Plant Resour..

[26] Zuo J., Qi T.L., Hu X.Y. (2023). Research Progress in Chemical Constituents and Modern Pharmacology of Poria Cocos. Acta Chin. Med. Pharmacol..

[27] Wang N., Gu X.Y., Wu Y., Wu H.Y., Liang X.Y., Xiu Y.F. (2021). Research overview of the clinical application and pharmacological action of Jineijin. Jiangsu J. Tradit. Chin. Med..

[28] Wang Z.Y., Liu J., Zhu Z., Su C.F., Sreenivasmurthy S.G., Iyaswamy A., Lu J.H., Chen G., Song J.X., Li M. (2021). Traditional Chinese medicine compounds regulate autophagy for treating neurodegenerative disease: A mechanism review. Biomed. Pharmacother..

[29] Ho Y.S., So K.F., Chang R.C. (2010). Anti-aging herbal medicine—How and why can they be used in aging-associated neurodegenerative diseases?. Ageing Res. Rev..

